# Screening the *Thermotoga maritima* genome for new wide-spectrum nucleoside and nucleotide kinases

**DOI:** 10.1016/j.jbc.2023.104746

**Published:** 2023-04-23

**Authors:** Katja F. Winkler, Lena Panse, Caroline Maiwald, Josefine Hayeß, Pascal Fischer, Maryke Fehlau, Peter Neubauer, Anke Kurreck

**Affiliations:** 1 Technische Universität Berlin, Faculty III Process Sciences, Institute of Biotechnology, Chair of Bioprocess Engineering, Berlin, Germany; 2 BioNukleo GmbH, Berlin, Germany

**Keywords:** *Thermotoga maritima*, nucleotide analogs, nucleoside/nucleotide kinase, nucleotide metabolism, ribokinase, pyruvate kinase, acetate kinase, pyruvate-phosphate-dikinase, nucleotidase, thermostable, substrate specificity

## Abstract

Enzymes from thermophilic organisms are interesting biocatalysts for a wide variety of applications in organic synthesis, biotechnology, and molecular biology. Next to an increased stability at elevated temperatures, they were described to show a wider substrate spectrum than their mesophilic counterparts. To identify thermostable biocatalysts for the synthesis of nucleotide analogs, we performed a database search on the carbohydrate and nucleotide metabolism of *Thermotoga maritima*. After expression and purification of 13 enzyme candidates involved in nucleotide synthesis, these enzymes were screened for their substrate scope. We found that the synthesis of 2′-deoxynucleoside 5′-monophosphates (dNMPs) and uridine 5′-monophosphate from nucleosides was catalyzed by the already known wide-spectrum thymidine kinase and the ribokinase. In contrast, no NMP-forming activity was detected for adenosine-specific kinase, uridine kinase, or nucleotidase. The NMP kinases (NMPKs) and the pyruvate-phosphate-dikinase of *T. maritima* exhibited a rather specific substrate spectrum for the phosphorylation of NMPs, while pyruvate kinase, acetate kinase, and three of the NMPKs showed a broad substrate scope with (2′-deoxy)nucleoside 5′-diphosphates as substrates. Based on these promising results, *Tm*NMPKs were applied in enzymatic cascade reactions for nucleoside 5′-triphosphate synthesis using four modified pyrimidine nucleosides and four purine NMPs as substrates, and we determined that base- and sugar-modified substrates were accepted. In summary, besides the already reported *Tm*TK, NMPKs of *T. maritima* were identified to be interesting enzyme candidates for the enzymatic production of modified nucleotides.

Natural and modified nucleotides are widely used as drugs to treat cancer or viral infections (1), food additives (2), or as reagents for molecular biology applications (3). Recently, they have contributed significantly to the containment of the Corona pandemic as building blocks of mRNA vaccines (4) or important components of PCR-based diagnostic kits (5).

Nucleotide analogs are mainly synthesized chemically. In contrast to the chemical synthesis routes, biocatalytic approaches offer several advantages: reactions can be performed at milder conditions, the use of harsh solvents is reduced, and the use of protection groups can be avoided due to a high regioselectivity and stereoselectivity (6, 7). For the biocatalytic nucleotide synthesis, enzymes of the *de novo* and salvage nucleotide pathway of various organisms were studied. These include, among others, phosphoribosyltransferases (PRTs), nucleoside kinases (NKs), nucleoside monophosphate kinases (NMPKs) and nucleoside diphosphate kinases (NDPKs). We recently developed a general approach for the one-pot synthesis of nucleoside triphosphates from nucleosides using nucleoside and nucleotide kinases (8). By coupling the phosphorylation reactions with an adenosine triphosphate (ATP) regeneration system, yields were significantly improved (8). Lately, cladribine triphosphate, a drug against leukemia and multiple sclerosis, was synthesized from 2-chloroadenine and phosphoribosyl pyrophosphate by an adenine phosphoribosyltransferase, polyphosphate kinase, and ribonucleotide reductase (9). In this cascade reaction, polyphosphates served as a low-cost phosphate donor.

Although enzymes from thermophilic organisms show many favorable characteristics like high thermal or solvent stability and broader substrate spectra compared to their mesophilic counterparts (10, 11, 12), to date only a few thermostable nucleoside or nucleotide phosphorylating enzymes were characterized (13). Enzymes accepting nucleosides or nucleoside diphosphates (NDPs) as substrates exhibited a broader substrate spectrum than NMPKs (Tables S1–S3). Interestingly, three classes of enzymes were described to phosphorylate nucleosides and exhibit a wide substrate spectrum: broad specificity NK (EC 2.7.1.B20), family B 6-phosphofructokinase (PfkB, EC 2.7.1.11), and thymidine kinases (TK, EC 2.7.1.21) (14, 15, 16, 17, 18). While TK predominantly accepted deoxyribonucleosides (Table S1), the NKs and PfkB preferred ribonucleosides (Table S1). An interesting finding for TK of *Thermotoga maritima* (*Tm*TK) was that it shows a wide substrate spectrum, which however differed depending on the reaction temperature applied (18). *Tm*TK accepted a wide range of modified nucleosides like 2′,3′-dideoxythymidine, 5-fluoro-2′-deoxyuridine, 2′,3′-dideoxy-3′-azidothymidine, 2′,3′-dideoxy-2′,3′-didehydrothymidine, 2′-fluoro-5-methyl-β-L-arabinofuranosyl-uracil, and dioxolane thymidine.

Inspired by the broad substrate scope of *Tm*TK, we studied the enzymes involved in nucleotide synthesis in the hyperthermostable bacterium *T. maritima* with the aim of identifying additional wide spectrum enzymes for biocatalytic applications. In total, 13 enzymes of *T. maritima* were expressed, purified, and characterized. The substrate scope of active enzymes was analyzed using a set of 26 natural (deoxy)nucleosides or nucleotides as substrates. Finally, we demonstrated the synthesis of base- and sugar-modified nucleotides in cascade reactions with *T. maritima* NMPKs. Thus, this study presents a set of thermostable *Tm*NMPKs suitable for the synthesis of natural and modified nucleotides.

## Results and discussion

### Identification of enzymes capable of phosphorylating nucleosides, NMPs, and NDPs in *T. maritima*

**Figure 1:**
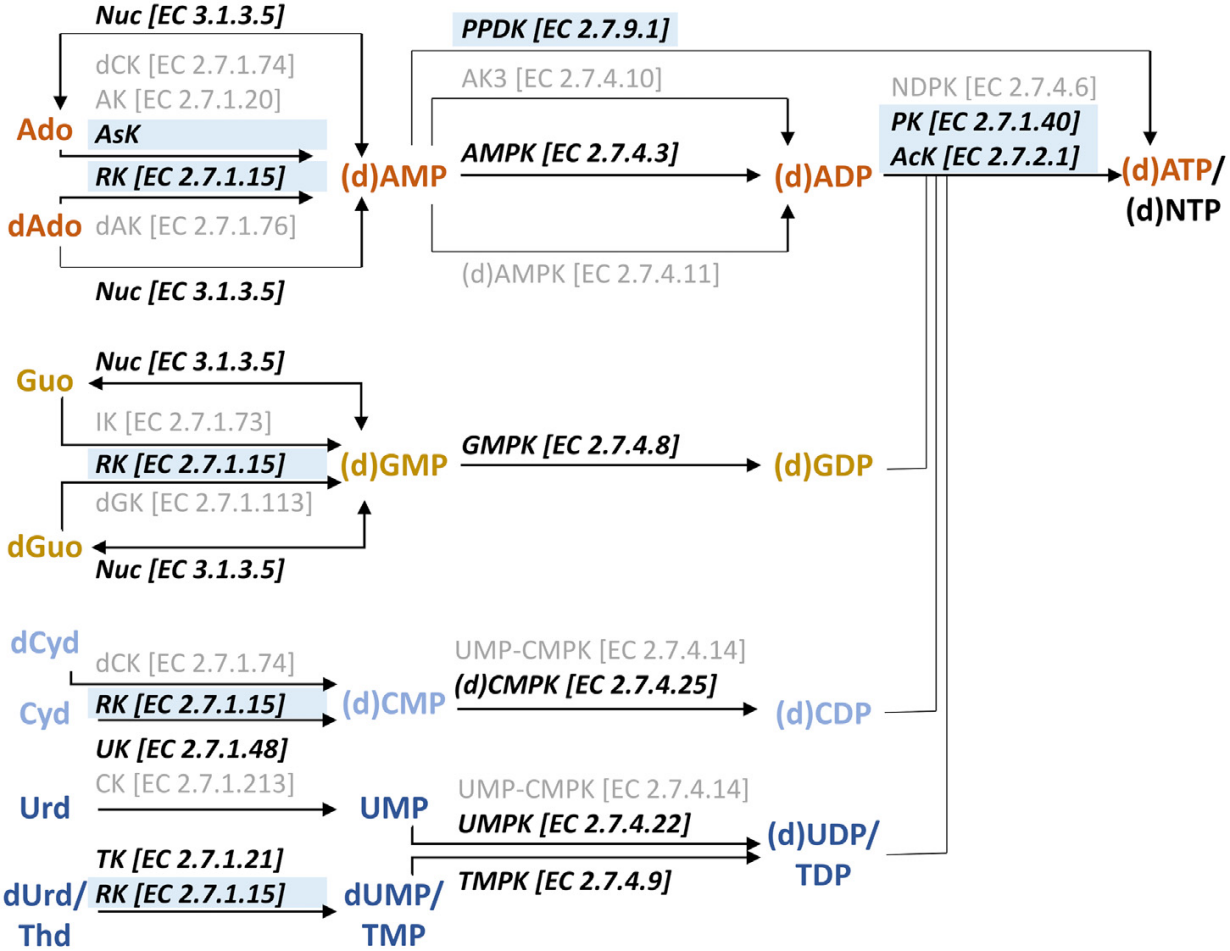
**Overview on enzymes potentially involved in the nucleotide salvage pathway of *T. maritima*.** Biocatalysts were identified on the one hand by analyzing the *T. maritima* specific KEGG pathway (*black font color*, *bold*, *italic*) in comparison to the corresponding reference pathway (*gray font color*). On the other hand, the *T. maritima* genome was screened for kinases described in the literature for accepting nucleosides or nucleotides as substrates (*blue background*). All enzymes marked in *bold* and *italics* were expressed, purified, and biochemically characterized in this study. Ado, adenosine; AK, adenosine kinase; AK3, isozyme 3 of adenylate kinase; AMPK, adenylate kinase; AsK, adenosine-specific kinase; CK, cytidine kinase; Cyd, cytidine dAdo, 2′-deoxyadenosine; (d)ADP, (2′-deoxy)adenosine 5′-diphosphate; dAK, deoxyadenosine kinase; (d)AMP, (2′-deoxy)adenosine 5′-monophosphate; dAMPK, deoxyadenylate kinase; (d)ATP, (2′-deoxy)adenosine 5′-triphosphate; (d)CDP, (2′-deoxy)cytidine 5′-diphosphate; dCK, deoxycytidine kinase; (d)CMP, (2′-deoxy)cytidine 5′-monophosphate; (d)CMPK, (deoxy)cytidylate kinase; dCyd, 2′-deoxycytidine; (d)GDP, (2′-deoxy)guanosine 5′-diphosphate; (d)GMP, (2′-deoxy)guanosine 5′-monophosphate;dGuo, 2′-deoxyguanosine; (d)NTP, (2′-deoxy)nucleoside 5′-triphosphate; (d)UDP, (2′-deoxy)uridine 5′-diphosphate; dUMP, 2′-deoxyuridine 5′-monophosphate; dUrd, 2′-deoxyuridine; GMPK, guanylate kinase; Guo, guanosine; IK, inosine kinase; NDPK, nucleoside diphosphate kinase; Nuc, nucleotidase; PK, pyruvate kinase; PPDK, pyruvate-phosphate-dikinase; RK, ribokinase; TDP, thymidine 5′-diphosphate; Thd, thymidine, TK, thymidine kinase; TMP: thymidine 5′-monophosphate; TMPK, deoxythymidylate kinase; UK, uridine kinase; UMP, uridine 5′-monophosphate; UMPK, uridylate kinase; Urd, uridine.

To identify enzymes of *T. maritima* involved in the phosphorylation of nucleosides, we analyzed both the Kyoto Encyclopedia of Genes and Genomes (KEGG) database and the *T. maritima* genome (Figs. 1 and S1). Next to the known *Tm*TK (18), in the genome an adenosine-specific kinase (*Tm*AsK) and a three-domain uridine kinase (*Tm*UK, annotated as NK in the genome sequence) were found. Adenosine-specific kinases are rarely studied, and the *Tm*UK shows an unusual protein sequence as it consists of a NK superfamily domain and a threonyl-tRNA synthetase domain (Fig. S2*A*). Neither a deoxycytidine kinase, a (deoxy)adenosine kinase, an inosine kinase, deoxyguanosine kinase, nor a deoxynucleoside kinase was found in *T. maritima* (Figs. 1 and S1).

As other enzyme classes like nucleotidases (19), acid phosphatases (20), PfkB (15, 16, 21), or polyphosphate kinases (22) are also known for the phosphorylation of nucleosides or nucleotides, they were also included in this study. Indeed, the KEGG pathway for the purine nucleotide metabolism suggests that the 5′/3′-nucleotidase SurE (*Tm*Nuc, also classified as acid phosphatase) of *T. maritima* (23) might catalyze the formation of purine NMPs (Fig. 1) which led us to integrate this enzyme into our study. Additionally, the *T. maritima* genome encodes for six enzymes of the PfkB-family including a ribokinase (24). Ribokinase of *T. maritima* (*Tm*RK) was described to show a high structural similarity to the NK of *Methanocaldococcus janaschii* (21) which accepted all natural ribonucleosides (15). Therefore, the *Tm*RK was selected in this study to evaluate whether it can accept nucleosides as substrates. Other PfkB-family enzymes were excluded from this study as they were shown before to be very specific for their sugar substrate (24).

For the second phosphorylation step, five NMPKs, namely an adenylate kinase (*Tm*AMPK), a guanylate kinase (*Tm*GMPK), an uridylate kinase (*Tm*UMPK), a deoxythymidylate kinase (*Tm*TMPK), and a (deoxy)cytidylate kinase (*Tm*(d)CMPK) were identified (Fig. 1). Additionally, in the genome, a pyruvate-phosphate-dikinase (*Tm*PPDK) was found. This enzyme was described before to catalyze the phosphorylation of nucleoside monophosphates directly to nucleoside triphosphates using phosphoenolpyruvate and pyrophosphate as substrates (25).

The conversion of NDPs to nucleoside 5′-triphosphates (NTPs) usually is catalyzed by wide-spectrum NDPKs. Surprisingly, the genome of *T. maritima* does not encode for a NDPK. Therefore, we included enzymes capable of performing a similar function as NDPKs in this study. We focused on enzymes widely used for ATP regeneration as they are well known to catalyze NTP formation. Furthermore, it was shown that pyruvate kinase is a candidate for a phosphoenolpyruvate (PEP)-dependent NDP kinase activity in *Escherichia coli* and *L. lactis* due to a wide substrate scope (26). Studying the *T. maritima* genome, an acetate kinase (*Tm*AcK) (27) and a pyruvate kinase (*Tm*PK) (28) were identified. A polyphosphate kinase, however, was not identified in the genome of *T. maritima* (29).

To explore whether the substrate specificities correlate with gene annotation, the 13 described enzymes putatively involved in the phosphorylation of nucleosides, NMPs, or NDPs (Fig. 1) were expressed, purified, and characterized in a next step.

### Expression and thermal characterization of the *T. maritima enzymes*

After gene synthesis, all *T. maritima* enzymes were successfully expressed in *E. coli* I^q^ cells and purified by affinity chromatography (Fig. S3). Protein yields were in the range of 0.8 to 15 mg g^−1^ cell pellet wet weight.

One advantage of thermostable enzymes is the possibility to use a heat treatment step to enhance protein purification. For *Tm*TK and *Tm*PK heat steps at 70 to 80 °C for 20 to 30 min were already described (18, 28). To confirm the thermostability of all expressed enzymes, we analyzed the heat denaturation of the enzymes purified by affinity chromatography in a thermal shift assay at pH values of 7, 8, and 9 in the absence and presence of 1,4-dithiothreitol (DTT). Indeed, no denaturation of the enzymes was detected up to 95 °C and during an additional 20 min incubation step at 95 °C (Fig. S4). Since the enzymes were shown to be hyperthermostable, they were re-expressed and purified by heat treatment and affinity chromatography.

### Activity testing of the *T. maritima* enzymes at varying pH

Having purified enzymes in hand, activity was determined at 37 °C using their putative natural nucleoside or nucleotide substrates. Phosphotransferase activity was confirmed for all enzymes except for *Tm*Nuc, *Tm*UK, and *Tm*AsK. As nucleosides are not the natural substrates for nucleotidase, additional activity tests were performed with adenosine 5′-nucleotides, and phosphatase activity toward adenosine 5′-monophosphate (AMP) (23) and adenosine 5′-diphosphate (ADP) was shown (data not shown). Hence, *Tm*Nuc is not able to phosphorylate nucleosides under the applied conditions.

Studies on adenosine-specific kinase (AsK) are rare. The first described enzyme was PAE2307, a protein from the hyperthermophilic archaeon *Pyrobaculum aerophilum* (30). Structural studies in combination with fluorescence spectroscopic analysis indicated a binding of adenosine and AMP to the adenosine-specific kinase like protein. Furthermore, a phosphorylation of the conserved histidine residue in the putative substrate-binding site was observed. Hence, it was suggested that the enzyme is a new class of adenosine kinase. Therefore, we evaluated if *Tm*AsK can phosphorylate adenosine. However, no AMP-forming activity was observed (data not shown). As biochemical and structural studies on a homologous enzyme of *Thermus thermophilus* indicated that the enzymatic function of the “adenosine-specific kinase” family is rather an ADP cleavage to AMP (31), we evaluated if the *Tm*AsK shows ADP phosphatase activity. However, no activity was observed using ADP as substrate for *Tm*AsK (data not shown). Additionally, no phosphatase activity was detected with AMP and ATP. Since MgCl_2_ was described to inhibit phosphatase activity of AsK (31), reactions were also performed without the addition of MgCl_2_ and DTT. However, no activity was detected as well (data not shown). Thus, neither a phosphotransferase nor a phosphatase activity was shown for *Tm*AsK under the conditions tested.

The *Tm*UK was described before to be a result of interkingdom gene fusions, which is shared by *Treponema pallidum* and *T. maritima* (32). Using the full-length protein, no UK activity was observed in this study (Fig. S2). However, ATP phosphatase activity was observed in the presence of uridine (Urd). Therefore, we decided to study if truncated versions (*Tm*UKs1-4, Fig. S2) only coding for the UK domain would show Urd phosphorylation activity. Truncated UK variants were cloned, heterologously expressed in *E. coli* and purified by affinity chromatography. While *Tm*UKs1-3 were produced in substantial amount and with good purity, *Tm*UKs4 could not be produced since only insoluble protein was obtained. Activity testing with Urd as substrate revealed no formation of uridine 5′-monophosphate (UMP) with all truncated UK variants. These results together with the observed ATP phosphatase activity in the presence of Urd fit well to the hypothesis that the three-domain UK of *T. pallidum* and *T. maritima* are involved in the autoregulation of translation (32).

For all enzymes showing phosphorylating activity toward nucleosides or nucleotides working pH ranges were determined, except for *Tm*TK, *Tm*PK, *Tm*AcK, and *Tm*PPDK. For the latter enzymes, the optimal reaction pH has already been described ((18, 25, 27, 28), Table 1). Working pH ranges differed depending on the enzyme classes. While *Tm*PK and *Tm*AcK preferred a lower reaction pH, *Tm*TK, *Tm*RK, and nearly all *Tm*NMPKs (except for *Tm*(d)CMPK) showed a comparable activity in a pH range of 7 to 9 (Table 1 and Fig. S5).

**Table 1:** Working pH range of the studied *T. maritima* enzymes involved in nucleotide synthesis

Enzyme	pH range	Reference
PfkB family		
*Tm*RK	7–9^a^	This study
Nucleoside kinases		
*Tm*TK	7–9	(18)
NMP kinases		
*Tm*AMPK	7–9^a^	This study
*Tm*(d)CMPK	7–8^a^	This study
*Tm*GMPK	7–9^a^	This study
*Tm*TMPK	7–9^a^	This study
*Tm*UMPK	7–9^a^	This study
*Tm*PPDK	7.0–7.5^b^	(25)
NDP kinases		
*Tm*PK	6.0^b^	(28)
*Tm*AcK	7.0^b^	(27)

a The working pH ranges were determined by comparing specific activities (Fig. S5). Reactions with 2 mM MgCl_2_, 5 mM DTT, 50 mM KCl, 1 mM substrate (AMPK: dAMP, (d)CMPK: CMP, GMPK: GMP, RK: Urd, TMPK: TMP, UMPK: UMP), and 1.2 mM ATP in 70 mM Tris-HCl (pH 7, 8, 9) were preheated to 37 °C. Reactions were started with appropriate enzyme dilutions. Samples were stopped after 5 min incubation with cold deionized water and were analyzed by the luminescent assay.

b Optimal pH.

### Evaluation of the substrate scope

To validate the substrate scope of the enzymes phosphorylating either nucleosides or nucleotides, reactions with 26 natural nucleosides or nucleotides were performed and analyzed by either thin-layer chromatography (TLC) or high-performance liquid chromatography (HPLC) after an incubation time of 19 h at 37 °C.

**Figure 2:**
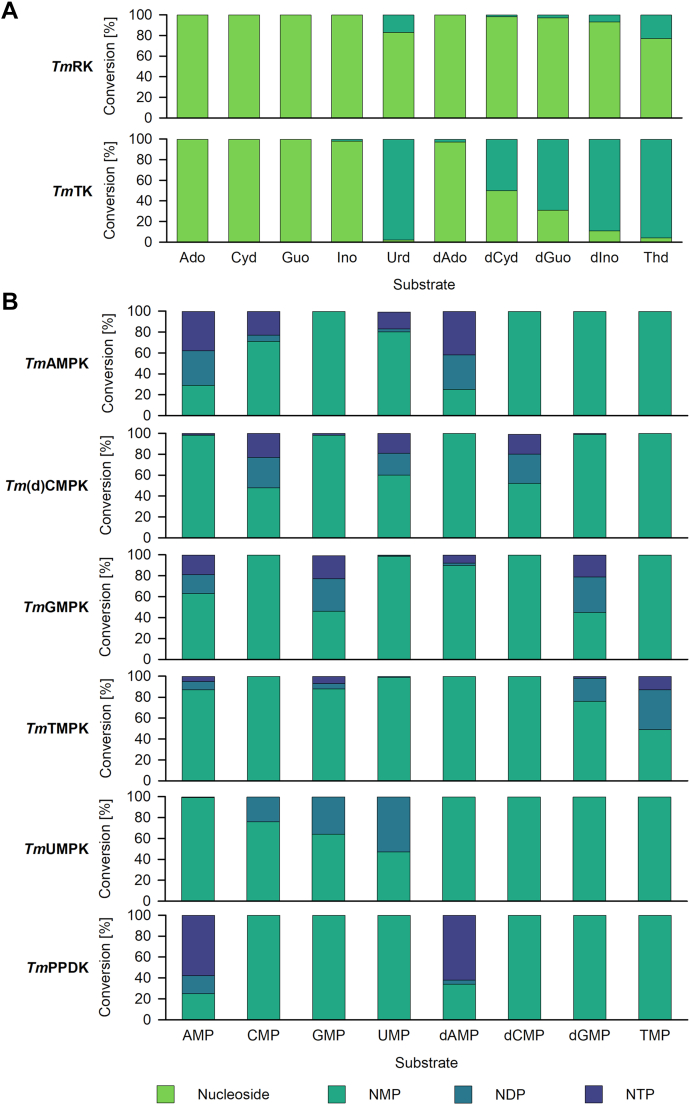
**Substratescope of*****Tm*****RK,*****Tm*****TK,*****Tm*****NMPKs and** ***Tm*****PPDK.** Reactions were performed under standard conditions: 70 mM Tris-HCl pH 7.6, 2 mM MgCl_2_, 5 mM DTT, 50 mM KCl, 1.2 mM phosphate donor and 1 mM nucleoside (*Tm*RK, *Tm*TK, *A*) or NMP (*Tm*NMPKs and *Tm*PPDK, *B)*. As phosphate donors ATP or dATP (for AMP reactions) were applied, except for *Tm*PPDK, where PEP and Na_4_O_7_P_2_ were used. Reactions were incubated for 19 h at 37 °C. Samples were analyzed by HPLC. Ado, adenosine; AMP, adenosine 5′-monophosphate; AMPK, adenylate kinase; CMP, cytidine 5′-monophosphate; Cyd, cytidine; dAdo, 2′-deoxyadenosine; dAMP, 2′-deoxyadenosine 5′-monophosphate; dATP, 2′-deoxyadenosine 5′-triphosphate; dCMP, 2′-deoxycytidine 5′-monophosphate; (d)CMPK, (deoxy)cytidylate kinase; dCyd, 2′-deoxycytidine; dGMP, 2′-deoxyguanosine 5′-monophosphate; dGuo, 2′-deoxyguanosine; dIno, 2′-deoxyinosine; DTT, 1,4-dithiothreitol; GMP, guanosine 5′-monophosphate; GMPK, guanylate kinase; Guo, guanosine; HPLC, high-performance liquid chromatography; Ino, inosine; NMPK, nucleoside monophosphate kinase; PEP, phosphoenolpyruvate; PPDK, pyruvate-phosphate-dikinase; RK, ribokinase; Thd, thymidine; TK, thymidine kinase; *Tm*, *Thermotoga maritima*; TMP, thymidine 5′-monophosphate; TMPK, deoxythymidylate kinase; UMP, uridine 5′-monophosphate; UMPK, uridylate kinase; Urd, uridine.

*Tm*TK was described to be specific for thymidine and uridine derivatives (18). In our study, *Tm*TK accepted all deoxynucleosides and uridine, with 2′-deoxyadenosine being the worst substrate (Fig. 2*A*).

The substrate spectrum of *Tm*RK with a set of sugars was studied before, and it was shown that the enzyme is very specific for D-ribose (24). Although *Tm*RK seems to be a rather specific sugar kinase, it phosphorylates the natural nucleosides thymidine and uridine as well as deoxyguanosine, deoxyinosine, and deoxycytidine albeit with lower conversion (Fig. 2*A*). The preference for pyrimidine nucleosides is in good accordance with *Pyrobaculum caldifontis* PfkB, which preferred cytidine and uridine (16). Interestingly, while other PfkB members preferred ribonucleosides (15, 16), *Tm*RK seems to prefer deoxynucleosides.

NMPKs of *T. maritima* showed a rather narrow substrate scope, except for *Tm*UMPK which accepted all natural riboNMPs (Fig. 2*B*). The other NMPKs accepted their preferred substrate next to a few additional substrates. As an example, *Tm*(d)CMPK accepted cytidine 5′-monophosphate (CMP), 2′-deoxycytidine 5′-monophosphate, and UMP.

**Figure 3:**
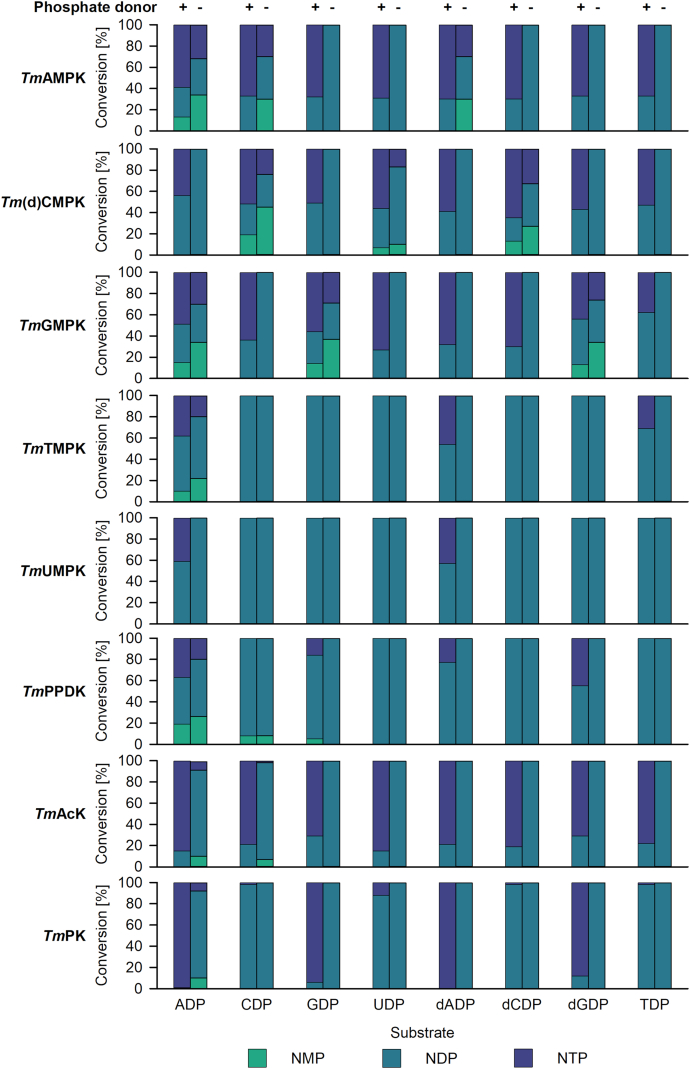
**Substrate scope of *Tm*NMPKs, *Tm*PPDK, *Tm*AcK, and *Tm*PK using NDPs as substrates.** Reactions were performed under standard conditions (70 mM Tris-HCl pH 7.6, 2 mM MgCl_2_, 5 mM DTT, 50 mM KCl, 1 mM substrate, 1.2 mM phosphate donor) either with (+) or without (−) phosphate donor. As phosphate donors ATP or dATP (for AMP reactions) for *Tm*NMPKs, PEP for *Tm*PK, AcetylP for *Tm*AcK, as well as PEP and Na_4_O_7_P_2_ for *Tm*PPDK were applied. Reactions were incubated for 19 h at 37 °C, and samples were analyzed by HPLC. AcK, acetate kinase; AcetylP, acetyl phosphate; ADP, adenosine 5′-diphosphate; AMPK, adenylate kinase; CDP, cytidine 5′-diphosphate; dADP, 2′-deoxyadenosine 5′-diphosphate; dATP, 2′-deoxyadenosine 5′-triphosphate; dCDP, 2′-deoxycytidine 5′-diphosphate; (d)CMPK, (deoxy)cytidylate kinase; dGDP, 2′-deoxyguanosine 5′-diphosphate; DTT, 1,4-dithiothreitol; GDP, guanosine 5′-diphosphate; GMPK, guanylate kinase; HPLC, high-performance liquid chromatography; NDP, nucleoside 5′-diphosphate; NMP, nucleoside 5′-monophosphate; NMPK, nucleoside monophosphate kinase; NTP, nucleoside 5′-triphosphate; PEP, phosphoenolpyruvate; PK, pyruvate kinase; PPDK, pyruvate-phosphate-dikinase; TDP, thymidine 5′-diphosphate; *Tm*, *Thermotoga maritima*; TMPK, deoxythymidylate kinase; UDP, uridine 5′-diphosphate; UMPK, uridylate kinase.

Interestingly, all NMPKs, except for UMPK, converted the NMP substrates to the respective NTPs with an NDP as intermediate (Fig. 3). Based on these results, we tested the ability of the NMPKs to accept NDPs as substrates with ATP as phosphate donor. Indeed, *Tm*AMPK, *Tm*GMPK, and *Tm*(d)CMPK were able to catalyze NTP formation with all tested natural (d)NDPs (Fig. 3). *Tm*TMPK and *Tm*UMPK catalyzed (d)ATP formation, and *Tm*TMPK additionally converted thymidine 5′-diphosphate to thymidine 5′-triphosphate. Thus, *T. maritima* NDPK deficiency might be compensated by *Tm*AMPK, *Tm*GMPK, and *Tm*(d)CMPK. NDPK activity of NMPKs has already been described before for diverse adenylate kinase (AMPKs) (33, 34, 35) and human UMP-CMPK (34). Human NMPKs were described to show real NDPK activity (NDP+ATP↔NTP+ADP) (34). In contrast, NDPK activity of bacterial AMPKs was explained by the commonly known reversibility of the NMPK reaction ($N_{1}MP+N_{2}TP\;↔N_{1}DP+N_{2}DP$) (33, 34, 35).

As for some reactions using NDPs as substrates and NTPs as phosphate donor, NMP formation was detected, we validated whether NMP formation is the result of the reverse NMPK reaction or based on basal NDP cleaving activity of the enzyme preparation. Reactions were performed with NDP as substrate, but without ATP. Indeed, with some substrates, NMPKs (except for UMPK) were able to catalyze the reaction 2NDP↔NMP+NTP. Accepted substrates correlated well with the NMP preference of the enzymes (Fig. 3). As an example, *Tm*(d)CMPK converted (d)CDP and UDP to the corresponding NMP and NTP.

*Tm*PPDK only converted AMP and 2′-deoxyadenosine 5′-monophosphate to the respective NTPs with (d)ADP formed as by-products to a lower percentage (Fig. 2*B*). Additionally, it was shown that (d)ADP and (d)GDP were substrates of *Tm*PPDK with comparable efficiency (Fig. 3). In good agreement with our results, *Tm*PPDK was described before to be highly specific for AMP when tested with ribonucleotide monophosphates (25). However, little information is available on the substrate scope of PPDKs since mainly reactions with AMP as substrate were reported. For *Clostridium symbiosum* PPDK, even an inhibitory effect of other NMPs like guanosine 5′-monophosphate (GMP) and inosine 5′-monophosphate was shown (36).

In contrast to *Tm*PPDK, *Tm*PK and *Tm*AcK accepted all natural riboNDPs and deoxyriboNDPs (Fig. 3). While *Tm*PK showed a preference for purine NDPs, *Tm*AcK converted all NDPs with comparable high conversion. Our results fit well to the observation that *Tm*AcK accepts ATP, guanosine 5′-triphosphate, inosine 5′-triphosphate, uridine 5′-triphosphate (UTP), and cytidine 5′-triphosphate as phosphate donors for acetyl phosphate (AcetylP) synthesis (27). PKs and acetate kinase (AcKs) are well known to show a wide substrate scope with PK and AcK of *Geobacillus stearothermophilus* being good examples. *Gs*PK was described to accept all natural riboNDPs ADP, guanosine 5′-diphosphate, inosine 5′-diphosphate, UDP and cytidine 5′-diphosphate, with the latter being the worst substrate (37). *Gs*AcK was studied in the direction of AcetylP synthesis, and ATP, guanosine 5′-triphosphate, UTP, and cytidine 5′-triphosphate were shown to serve as phosphate donors (38). Purine NTPs were preferred over pyrimidine NTPs.

In summary, *T. maritima* is able to synthesize all natural 5'-(d)NTPs starting from the respective (d)NMPs, although it does not possess a NDPK (Fig. S1). The majority of NMPKs as well as *Tm*AcK and *Tm*PK are able to phosphorylate NDPs to NTPs. Furthermore, *Tm*PPDK either converts (d)AMP to (d)ATP or (d)ADP and 2′-deoxyguanosine 5′-diphosphate to the corresponding (d)NTPs. Due to the presence of the wide-spectrum TK and the RK, phosphorylation of deoxynucleosides and uridine to the respective monophosphates is possible. A nucleoside kinase for the synthesis of riboNMPs, however, seems not to be present in *T. maritima* (Fig. S1). Though, in purine metabolism, enzymes of the complete *de novo* pathway are present, so that the synthesis of AMP and GMP is possible *via* this route (Fig. S1). In addition, the genome of *T. maritima* also contains PRT for adenine and hypoxanthine which catalyze the synthesis of AMP and GMP starting from the corresponding bases. However, based on the available data, the synthesis of CMP starting from cytidine or cytosine is not possible in *T. maritima*, since corresponding enzymes are missing (Fig. S1). Though, probably the salvage of CMP is catalyzed *via Tm*Nuc or deamination to uridine by cytidine deaminase.

### Enzymatic cascade reaction to synthesize 5′-NTP analogs

**Figure 4:**
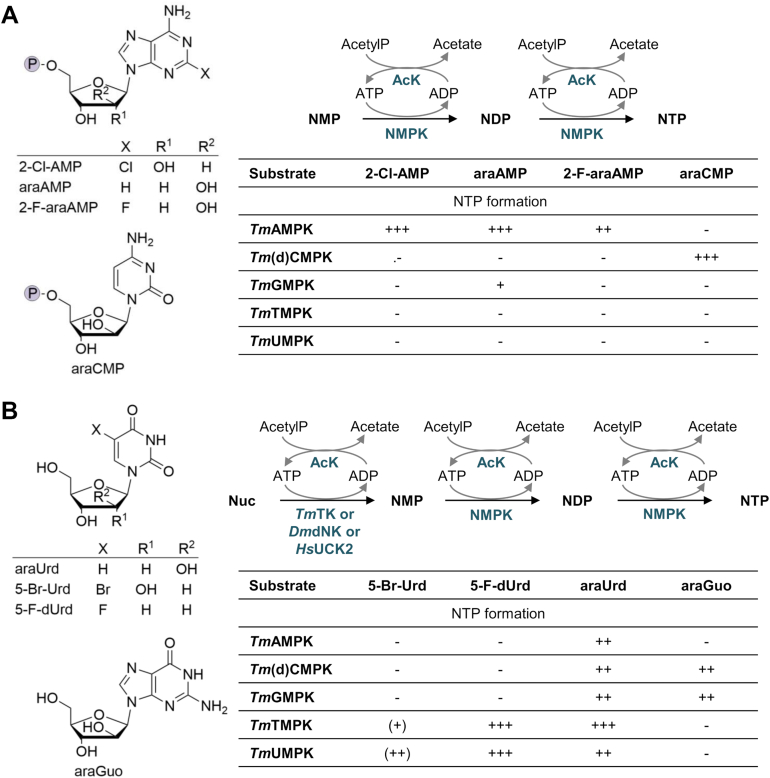
**Enzyme cascade reactions for the synthesis of 5′-nucleotide analogs.***A* and *B*, enzyme cascade reactions were performed with 10 mM MgCl_2_, 5 mM DTT, 50 mM KCl, 1 mM NMP (*A*) or nucleosides (*B*), 1.2 mM ATP, 5 mM AcetylP in 70 mM Tris-HCl pH 7.6. Enzymes were applied with 0.02 mg ml^−1^ NK (*Hs*UCK2: 5-Br-Urd, others: *Dm*dNK) 0.04 mg ml^−1^ NMPK and 0.02 mg ml^−1^ AcK. Cascade reactions were incubated for 19 h at 37 °C and analyzed by TLC or HPLC (see Figs. S6 and S7). (+) <10% NDP formation, (++) NDP formation between 10% and 50%, + <10% NTP formation, ++ NTP formation between 10% and 50%, +++ NTP formation >50%. 2-Cl-AMP, 2-chloro-adenosine 5′-monophosphate; 2-F-araAMP, fludarabine phosphate; 5-Br-Urd, 5-bromo-uridine; 5-F-dUrd, 5-flouro-2′-deoxyuridine; AcK, acetate kinase; AcetylP, acetyl phosphate; ADP, adenosine 5′-diphosphate; AMPK, adenylate kinase; araAMP, vidarabine phosphate; araCMP, cytarabine phosphate; araGuo, arabinofuranosylguanine; CMPK, (deoxy)cytidylate kinase; *Dm*dNK, *Drosophila melanogaster* deoxynucleoside kinase; DTT, 1,4-dithiothreitol; GMPK, guanylate kinase; HPLC, high-performance liquid chromatography; *Hs*UCK2, human uridine-cytidine kinase 2; NDP, nucleoside 5′-diphosphate; NMPK, nucleoside monophosphate kinase; *Tm*, *Thermotoga maritima*; TMPK, deoxythymidylate kinase; UMPK, uridylate kinase.

With the initial aim in mind to produce 5′-NTP analogs, we evaluated whether *Tm*NMPKs accept nucleotide analogs as substrates (Figs. 4, S6 and S7). Therefore, enzyme reactions with ATP as phosphate donor were run for 19 h at 37 °C, and samples were either analyzed by TLC or HPLC. The NMP analogs 2-chloro-AMP, vidarabine phosphate, fludarabine phosphate, and cytarabine phosphate (araCMP) were tested as substrates for the *Tm*NMPKs. As NMPs were not available for pyrimidine analogs and for arabinofuranosylguanine, we screened the *Tm*NMPKs in cascade reactions with the wide spectrum NKs *Drosophila melanogaster* deoxynucleoside kinase (*Dm*dNK) or human uridine-cytidine kinase 2 (*Hs*UCK2, NK15) for the phosphorylation of 5-bromouridine, 5-fluoro-2′-deoxyuridine, arabinofuranosyluracil, and arabinofuranosylguanine. To enhance the synthesis of the NTPs (8), an ATP regeneration system based on *Tm*AcK was integrated.

All modified substrates tested were at least accepted by one *Tm*NMPK (Figs. 4, S6 and S7), which indicates their high potential for the *in vitro* synthesis of nucleotide analogs. So far, mainly NMPKs from human and pathogenic organisms were studied for their ability to phosphorylate nucleotide analogs, since they are responsible for the therapeutic efficiency of nucleoside/nucleotide drugs (39, 40, 41, 42). Apart from that, there are only few reports about nucleotide analog synthesis involving NMPKs, including the synthesis of 3′-F-(d)ATP and 3′F-(d)GTP by yeast NMPKs (43), several 2′-/3′-modified NMPs by calf thymus NMPKs (44), 5-F-CTP, 5-F-UTP by *E. coli* NMPKs (45), as well as adenosine 5′-(*O*-1-thiotriphosphate), adenosine 5′-(*O*-2-thiotriphosphate), and ribavirin triphosphate by rabbit muscle AMPK (46, 47). Notably, to our best knowledge, we demonstrated here for the first time the acceptance of nucleotide analogs by thermostable NMPKs.

For *Tm*AMPK, NTP formation was observed for all AMP analogs (Fig. 4). Interestingly, although CMP and UMP are natural substrates, araUMP was accepted but not araCMP. Furthermore, neither the other UMP analogs nor araGMP were phosphorylated. The preference of AMP analogs is in good agreement with the data for other AMPKs (8, 48, 49). In contrast to the phosphorylation of deoxycytidine analogs like araCMP (48), the acceptance of araUMP was not described before for AMPKs.

*Tm*(d)CMPK converted the sugar-modified substrates araCMP, araGMP, and araUMP, but not the AMP analogs and the base-modified UMP analogs (Fig. 4). Besides araCMP, 2′-3′-dideoxy-CMP was a substrate for *E. coli* CMPK (50). Whereas the *E. coli* CMPK accepted sugar- and base-modified analogs of pseudouridine monophosphate (51), *Tm*CMPK was not phosphorylating the 5-halogenated UMP analogs.

*Tm*GMPK phosphorylated the sugar-modified NMPs araGMP, vidarabine phosphate, and araUMP (Fig. 4). However, neither the base-modified adenosine nor the uridine analogs were substrates of *Tm*GMPK. Guanylate kinase (GMPKs) were described before to accept guanosine analogs like araGMP and 2′-2′-difluorodeoxyguanosine monophosphate for *Dm*GMPK (52), as well as 8-azaGMP, ganciclovir, and acyclovir for human erythrocyte GMPK (53, 54, 55).

In contrast to the other three *Tm*NMPKs, all uridine analogs were accepted by *Tm*TMPK and *Tm*UMPK (Fig. 4). Notably, for 5-Br-UMP only the diphosphate was detected. This might be explained by the substrate scope observed for both NMPKs. Neither of the two enzymes was able to convert UDP to UTP (Fig. 3). Interestingly, for the other uridine analogs, the triphosphate was formed. Thus, *Tm*AcK very likely accepted the modified uridine diphosphates, except for 5-Br-UDP, as substrates.

In summary, we demonstrated the ability of the *T. maritima* NMPKs to phosphorylate base- and sugar-modified substrates in enzymatic cascade reactions. Thus, they are interesting thermostable biocatalysts for the *in vitro* synthesis of modified 5′-nucleotides.

## Conclusions

In this study, we investigated 13 enzymes of *T. maritima* for their ability to phosphorylate natural and modified nucleosides or nucleotides. Three of the enzymes, namely *Tm*AsK, *Tm*UK, and *Tm*Nuc, did not show phosphotransferase activity with ATP as phosphate donor under the conditions tested. Broad substrate spectra toward natural substrates were shown for *Tm*TK, *Tm*PK, and *Tm*AcK, whereas *Tm*PPDK and *Tm*RK were more specific. While all five *Tm*NMPKs had a narrow substrate scope toward NMPs, we detected a broad NDPK activity for three *Tm*NMPKs. In addition, we demonstrated for the first time the phosphorylation of base- and sugar-modified NMPs by thermostable NMPKs in enzymatic cascade reactions. While we focused on modifications in the 2′-position of the ribosyl residue or the 5-position of the base, the synthesis of further modifications can be analyzed in the future. Of particular interest here could be 3′- or 4′-modified nucleotides or C-nucleotides. Thus, our work lays the foundation for the application of these thermostable enzymes for the biocatalytic production of nucleotide analogs.

## Experimental procedures

### General information

All chemicals and solvents were of analytical grade or higher and purchased from Sigma-Aldrich, Carl Roth, TCI Deutschland, Carbosynth, or VWR. Nucleosides and nucleotides were acquired from Alfa Aesar, Carl Roth, Sigma-Aldrich, Carbosynth Limited, and TCI. Stock solutions with concentrations of 10 and 50 mM were prepared in deionized water, and aliquots were stored at −20 °C.

The human uridine-cytidine kinase 2 (*Hs*UCK2, NK15) was kindly provided by BioNukleo GmbH and stored at −20 °C.

### Identification of enzymes involved in the nucleotide salvage pathway of *T. maritima*

The KEGG pathway of *T. maritima* in comparison to the reference pathway was analyzed (purine metabolism: tma00230 and pyrimidine metabolism: tma00240). All enzymes involved in the nucleotide pathway were chosen for gene synthesis. Furthermore, the genome of *T. maritima* (accession number: NC_023151.1) was analyzed for proteins being able to phosphorylate either nucleosides, NMPs, or NDPs.

### Expression and purification of the *T. maritima* enzymes

Genes of interest were synthesized by GeneArt (ThermoFisher). Enzymes were expressed in *E. coli* I^q^ (NEB) using EnPresso B medium (Enpresso) based on the manufacturer’s recommendations. Briefly, a 50 ml main culture was inoculated either from a fresh transformation plate or an LB preculture. Protein expression was induced by the addition of 200 μM IPTG. Cells were harvested by centrifugation and stored at −20 °C until further use. For purification, the cell pellet was resuspended in binding buffer (50 mM NaH_2_PO_4_, 300 mM NaCl, 10 mM imidazole, pH 8), including 0.1 mM phenylmethylsulfonyl fluoride, 1 mg ml^−1^ lysozyme, 1 mM MgCl_2_, and 0.6 mg ml^−1^ DNase. After incubation for 30 min at room temperature, cells were disrupted by sonification and prepurified by heat treatment for 20 min at 80 °C. After centrifugation, the clear supernatant was loaded onto 0.5 to 2 ml Ni-NTA agarose (Jena Bioscience) columns. The columns were washed four times with 2 column volumes of washing buffer (binding buffer with 20 mM imidazole) and six times with 0.5 column volumes of elution buffer (binding buffer with 250 mM imidazole). The buffer of the elution fractions was exchanged by dialysis to 50 mM Tris-HCl pH 7.6, and enzymes were stored in 50% (v/v) glycerol at −20 °C.

The purity of the enzyme preparations was analyzed by SDS-PAGE. Protein concentration was determined by A280 measurements at a ThermoFisher Scientific NanoDrop One using the molar extinction coefficient E1% predicted by Protparam.

### Expression and purification of the *D. melanogaster* deoxynucleoside kinase

The *D. melanogaster* deoxynucleoside kinase (*Dm*dNK) plasmid was kindly provided by Prof. Birgitte Munch-Petersen (Roskilde University). The GST-fusion protein was produced as described before (8, 56) in *E. coli* BL21. The purified and tag-free enzyme was stored in 50% glycerol, 1% Triton X-100 and 1 mM DTT at −20 °C.

### Thermal shift assay

The melting point was analyzed as described before (57). In a total volume of 25 μl, 0.2 g l^−1^ enzyme and 5× SYPRO orange were incubated in 70 mM Tris-HCl pH 7, pH 8, or pH 9 with 0 or 5 mM DTT. The sealed PCR plate was incubated at 50 °C for 30 s and then heated in steps of 0.5 °C per 5 s to a final temperature of 95 °C in a Bio-Rad CFX96 Real-Time system. Fluorescence was measured at λex = 470 nm and λem = 570 nm. To estimate protein stability at elevated temperatures, proteins were further incubated at 95 °C for 20 min. Fluorescence was measured again afterward. The melting point was determined as the inflection point of the fluorescence intensity over the temperature.

### Enzyme activity assays

Enzyme reactions were performed in 50 to 200 μl volumes. Standard reaction mixtures consisted of 70 mM Tris-HCl pH 7.6, 2 mM MgCl_2_, 5 mM DTT, 50 mM KCl, 1 mM substrate (nucleoside, NMP or NDP), and 1.2 mM phosphate donor (ATP or 2′-deoxyadenosine 5′-triphosphate: NKs, NMPKs, RK; PEP: PK, PPDK; AcetylP: AcK) and 0.1 mg ml^−1^ enzyme. For the PPDK additionally, 1 mM sodium pyrophosphate (Na_4_O_7_P_2_) was applied.

Nucleoside phosphotransferase activity of Nuc was further tested as described before (19). Briefly, reactions including 1 mM 2′-deoxyinosine 2 mM inosine 5′-monophosphate, 20 mM MgCl_2_ and 4.5 mM ATP, and 0.1 mg ml^−1^ Nuc in 100 mM Tris-HCl pH 7.6 were performed. Additionally, reactions including 2 mM MgCl_2_, 5 mM DTT, 1 mM inosine, 1.2 mM GMP, 1.2 mM ATP, and 0.1 mg ml^−1^ enzyme in 70 mM Tris-HCl pH 7.6 were analyzed.

To analyze the nucleotide phosphatase activity of AsK and Nuc, the nucleotides AMP, ADP, and ATP were applied as substrates in the standard reaction mixture without an additional phosphate donor. AsK reactions were also performed without the addition of MgCl_2_ and/or DTT.

Reactions were incubated for 19 h at 37 °C and validated by TLC or HPLC.

Enzyme cascade reactions were performed with 10 mM MgCl_2_, 5 mM DTT, 50 mM KCl, 1 mM substrate (nucleoside or nucleotide), 1.2 mM ATP, and 5 mM AcetylP in 70 mM Tris-HCl pH 7.6. Enzymes were applied with 0.02 mg ml^−1^ NK, 0.04 mg ml^−1^ NMPK, and 0.02 mg ml^−1^ AcK. Cascade reactions were incubated for 19 h at 37 °C and analyzed by TLC or HPLC.

### Determination of the reaction pH

The influence of the reaction pH was evaluated by applying 70 mM Tris-HCl pH 7, 8, and 9. The following substrates were used: Urd: RK, 2′-deoxyadenosine 5′-monophosphate: AMPK, CMP: (d)CMPK, GMP: GMPK, TMP: TMPK, and UMP: UMPK. The reactions were preheated to 37 °C and started by the addition of diluted enzyme stock solution. After 5 min, a 30 μl sample was stopped with 780 μl cold deionized water and analyzed by the luminescent assay.

### Thin-layer chromatography (TLC)

After the reactions were stopped by freezing, 5 μl were spotted on a TLC plate. The running buffer consisted of deionized water, 25% ammonia, isopropanol and dioxan in a ratio of 1:4:5:0 (screening) or 4:3:2:4 (cascade reactions). The reaction compounds were identified by comparison to authentic standards under UV-light.

### High-performance liquid chromatography

For HPLC analysis, samples were stopped with ice-cooled deionized water and freeze-thawed. After centrifugation (21,500*g*, 4 °C, 15 min), samples were analyzed with a KNAUER Azura or Agilent 1200 system using a Phenomenex (Aschaffenburg) reversed phase Kinetex EVO C18 column (250 × 4.6 mm) as previously described (8, 58). Briefly, samples were measured at 260 nm and 34 °C with a flow rate of 1 ml min^−1^. Isocratic elution was performed using 80% A (0.1 M KH_2_PO_4_/K_2_HPO_4_, 8 mM tetrabutylammonium bisulfate, pH ca. 5.4) and 20% B (70% A, 30% MeOH) for 4 min followed by a gradient to 40% A and 60% B over 10 min, to 38% A and 62% B in 12 min, and back to 80% A and 20% B in 0.5 min. Initial conditions were maintained for 2.5 min.

Enzyme cascade reactions were analyzed using an adapted gradient: after an isocratic step of 80% A and 20% B for 4 min, 100% B was obtained in 16 min and maintained for 10 min. Then, initial conditions were restored in 0.5 min and maintained for 9.5 min.

Typical retention times were as follows: AMP: 7.5 min, ADP: 14.6 min, ATP: 19.6 min. Substrates and products were identified based on their retention time and UV absorption spectra.

Conversions were calculated according to (1) as the ratio between the peak area of compound X (P_X_) and the sum of all peak areas of the substrate and product(s) in the reaction (P_total_).

(1) $$Conversion\left(X\right)\left[\%\right]=100\times \frac{P_{X}}{P_{total}}$$

### Luminescent assay

The luminescent assay was performed as previously described (58). Briefly, the luminescence of 90 μl sample and 10 μl Kinase-Glo Max reagent (Promega) was measured by a Tecan infinite M1000 plate reader. The product formation was calculated from the remaining ATP in the reaction according to formula (2), where ${Lum}_{R}$ is the average luminescence signal of the reaction sample, and ${Lum}_{NC}$ is the average luminescence signal of the negative control.

(2) $$Product\left[\%\right]=100-\left(100\times \frac{{Lum}_{R}}{{Lum}_{NC}}\right)$$

## Data availability

All data depicted visually in the items and in the main text as well as in the Supplementary Material are available on request from the corresponding author.

## Supporting information

This article contains supporting information (Figures S1-S7; Tables S1-S3; and Refs. (14, 15, 16, 17, 18, 57, 59, 60, 61, 62, 63, 64, 65, 66, 67, 68, 69, 70, 71, 72, 73, 74)).

## Conflict of interest

A. K. is CEO of the biotech company BioNukleo GmbH. M. F. and C. M. are scientists at BioNukleo GmbH, and P. N. is a member of the advisory board. The authors declare no conflict of interest.
