# Supplementary material for: Screening the Thermotoga maritima genome for new wide-spectrum nucleoside and nucleotide kinases
Source: J Biol Chem. 2023 Apr 23;299(6):104746. doi: 10.1016/j.jbc.2023.104746 (PMC10248530; doi:10.1016/j.jbc.2023.104746)
Supplement: Supplemental Figures S1–S7 and Tables S1–S3 [file mmc1.docx]

**Supporting Information**

**Screening the *Thermotoga maritima* genome for new wide-spectrum nucleoside and nucleotide kinases**

Katja F. Winkler ^1^, Lena Panse ^1^, Caroline Maiwald ^2^, Josefine Hayeß ^1^, Pascal Fischer ^1^, Maryke Fehlau ^1,2^, Peter Neubauer ^1^ and Anke Kurreck ^1,2,^*

^1^ Technische Universität Berlin, Faculty III Process Sciences, Institute of Biotechnology, Chair of Bioprocess Engineering, Ackerstraße 76, 13355 Berlin, Germany

^2^ BioNukleo GmbH, Ackerstraße 76, 13355 Berlin, Germany

***** Correspondence: anke.wagner@tu-berlin.de

| **Table of content**  **Table S1.** Substrate scope of thermostable enzymes involved in the phosphorylation of natural nucleosides | 2 |
| --- | --- |
| **Table S2.** Substrate scope of thermostable enzymes involved in the phosphorylation of natural nucleoside monophosphates | 3 |
| **Table S3.** Substrate scope of thermostable enzymes capable to phosphorylate natural nucleoside diphosphates | 4 |
| **Figure S1** Synthesis of natural (deoxy)nucleotides in *T. maritima*. Purine (A) and pyrimidine (B) metabolism are shown. | 5 |
| **Figure S2.** Construction and activity validation of truncated *Tm*UK variants | 6 |
| **Figure S3.** Purified enzymes of *T. maritima* putatively involved in the phosphorylation of nucleosides or nucleotides | 7 |
| **Figure S4.** Melting point analysis for the 13 *T. maritima* enzymes putatively involved in the phosphorylation of nucleosides or nucleotides. | 8 |
| **Figure S5.** pH dependency of the NMPKs and RK of *T. maritima*. | 9 |
| **Figure S6.** Enzymatic cascade reactions to produce sugar- or base modified 5´-NTPs starting from the NMP analog. | 10 |
| **Figure S7.** Enzymatic cascade reactions to produce sugar- or base modified 5´-NTPs starting from the nucleoside analog | 11 |
| References | 12 |

**Table S1.** Substrate scope of thermostable enzymes involved in the phosphorylation of natural nucleosides.

| **enzyme** | **Ado** | **dAdo** | **Cyd** | **dCyd** | **Guo** | **dGuo** | **Ino** | **dIno** | **Urd** | **dUrd** | **Thd** | **Xan** |
| --- | --- | --- | --- | --- | --- | --- | --- | --- | --- | --- | --- | --- |
| **PfkB** | | | | | | | | | | | | |
| *Aeropyrum pernix* | [1],[2] |  | [2] |  | [2] |  | [2] |  | [2] |  | [2] | [2] |
| *Desulfurcoccus amylolyticus* | [2] |  | [2] |  | [2] |  | [2] |  | [2] |  | [2] | [2] |
| *Pyrobaculum calidifontis* | [3] | [3] | [3] | [3] | [3] | [3] | [3] |  | [3] | [3] |  | [3] |
| **nucleoside kinase** | | | | | | | | | | | | |
| *Methanocaldcoccus jannaschii* | [2] | [2] | [2] |  | [2] |  | [2] |  | [2] |  | [2] | [2] |
| *Thermoplasma acidophilum* | [4] | [4] | [4] |  | [4] |  | [4] |  | [4] |  | [4] |  |
| **cytidine kinase** | | | | | | | | | | | | |
| *Phorcysia thermohydrogeniphila* | [5] |  | [5] |  |  |  | [5] |  | [5] |  |  |  |
| *Thermovibrio guaymasensis* | [5] |  | [5] |  |  |  | [5] |  | [5] |  |  |  |
| **thymidine kinase** | | | | | | | | | | | | |
| *Rhodothermus marinus* |  |  |  |  |  |  |  |  |  |  | [6] |  |
| *Thermotoga maritima* |  | [7] |  | [7] |  | [7] |  |  | [7] | [7] | [7] |  |
| **uridine-cytidine kinase** | | | | | | | | | | | | |
| *Thermus thermophiles* | [8] | [8] | [8],[9] | [8] | [8] | [8] |  |  | [8] |  | [8] |  |

Abbreviations: Ado: adenosine, Cyd: cytidine, dAdo: 2´-deoxyadenosine, dCyd: 2´-deoxycytidine, dGuo: 2´-deoxyguanosine, dIno: 2´-deoxyinosine, dUrd: 2´-deoxyuridine, Guo: guanosine, Ino: inosine, PfkB: Family B 6-phosphofructokinase, Thd: thymidine, Urd: uridine, Xan: xanthosine. green: relative specificity ≥ 0.5 compared to the favorite substrate, yellow: 0.01 < relative specificity < 0.5 compared to the favorite substrate, turquoise: relative specificity ≤ 0.01 compared to the favorite substrate or described as negligible activity, purple: relative specificity not determined, blue: no substrate

**Table S2.** Substrate scope of thermostable enzymes involved in the phosphorylation of natural nucleoside monophosphates.

| **enzyme** | **AMP** | **dAMP** | **CMP** | **dCMP** | **GMP** | **dGMP** | **IMP** | **dIMP** | **UMP** | **dUMP** | **TMP** | **XMP** |
| --- | --- | --- | --- | --- | --- | --- | --- | --- | --- | --- | --- | --- |
| **adenylate kinase** | | | | | | | | | | | | |
| *Methanocaldcoccus* | [10–12] |  |  |  |  |  |  |  |  |  |  |  |
| *Thermotoga neapolitana* | [13] |  |  |  |  |  |  |  |  |  |  |  |
| **cytidylate kinases** | | | | | | | | | | | | |
| *Thermus thermophiles* |  |  | [14] |  |  |  |  |  |  |  |  |  |
| **thymidylate kinase** | | | | | | | | | | | | |
| *Aquifex aeolicus* | [15] |  | [15] |  | [15] |  |  |  | [15] |  | [15] |  |
| *Thermus thermophilus* |  |  |  |  |  |  |  |  |  |  | [16] |  |
| *Sulfolobus tokodaii* | [17] |  | [17] |  |  | [17] |  |  | [17] |  | [17] |  |
| **uridylate kinase** | | | | | | | | | | | | |
| *Pyrococcus furiosus* | [18] |  | [18] |  | [18] |  |  |  | [18] |  | [18] |  |
| *Sulfolobus solfataricus* | [19] |  | [19] |  | [19] |  | [19] |  | [19] | [19] | [19] | [19] |

Abbreviations: AMP: adenosine 5´-monophosphate, CMP: cytidine 5´-monophosphate, dAMP: 2´-deoxyadenosine 5´-monophosphate, dCMP: 2´-deoxycytidine 5´-monophosphate, dGMP: 2´-deoxyguanosine 5´-monophosphate, dIMP: 2´-deoxyinosine 5´-monophosphate, dUMP: 2´-deoxyuridine 5´-monophosphate, GMP: guanosine 5´-monophosphate, IMP: inosine 5´-monophosphate, TMP: thymidine 5´-monophosphate, UMP: uridine 5´-monophosphate, XMP: xanthosine 5´-monophosphate. green: relative specificity ≥ 0.5 compared to the favorite substrate, yellow: 0.01 < relative specificity < 0.5 compared to the favorite substrate, turquoise: relative specificity ≤ 0.01 compared to the favorite substrate or described as negligible activity, purple: relative specificity not determined, blue: no substrate

**Table S3.** Substrate scope of thermostable enzymes capable to phosphorylate natural nucleoside diphosphates.

| **enzyme** | **ADP** | **dADP** | **CDP** | **dCDP** | **GDP** | **dGDP** | **IDP** | **dIDP** | **UDP** | **dUDP** | **TDP** |
| --- | --- | --- | --- | --- | --- | --- | --- | --- | --- | --- | --- |
| **nucleoside diphosphate kinases** | | | | | | | | | | | |
| *Thermus thermophilus* |  |  | [20] |  | [20] |  |  |  |  |  |  |
| *Pyrobaculum aerophilum[a]* |  | [21] |  | [21] |  | [21] |  |  |  |  | [21] |

[a] The reaction ADP + NTP was tested. Abbreviations: ADP: adenosine 5´-diphosphate, CDP: cytidine 5´-diphosphate, dADP: 2´-deoxyadenosine 5´-diphosphate, dCDP: 2´-deoxycytidine 5´-diphosphate, dGDP: 2´-deoxyguanosine 5´-diphosphate, dIDP: 2´-deoxyinosine 5´-diphosphate, dUDP: 2´-deoxyuridine 5´-diphosphate, GDP: guanosine 5´-diphosphate, IDP: inosine 5´-diphosphate, TDP: thymidine 5´-diphosphate, UDP: uridine 5´-diphosphate. green: relative specificity ≥ 0.5 compared to the favorite substrate, yellow: 0.01 < relative specificity < 0.5 compared to the favorite substrate, turquoise: relative specificity ≤ 0.01 compared to the favorite substrate or described as negligible activity, purple: relative specificity not determined, blue: no substrate


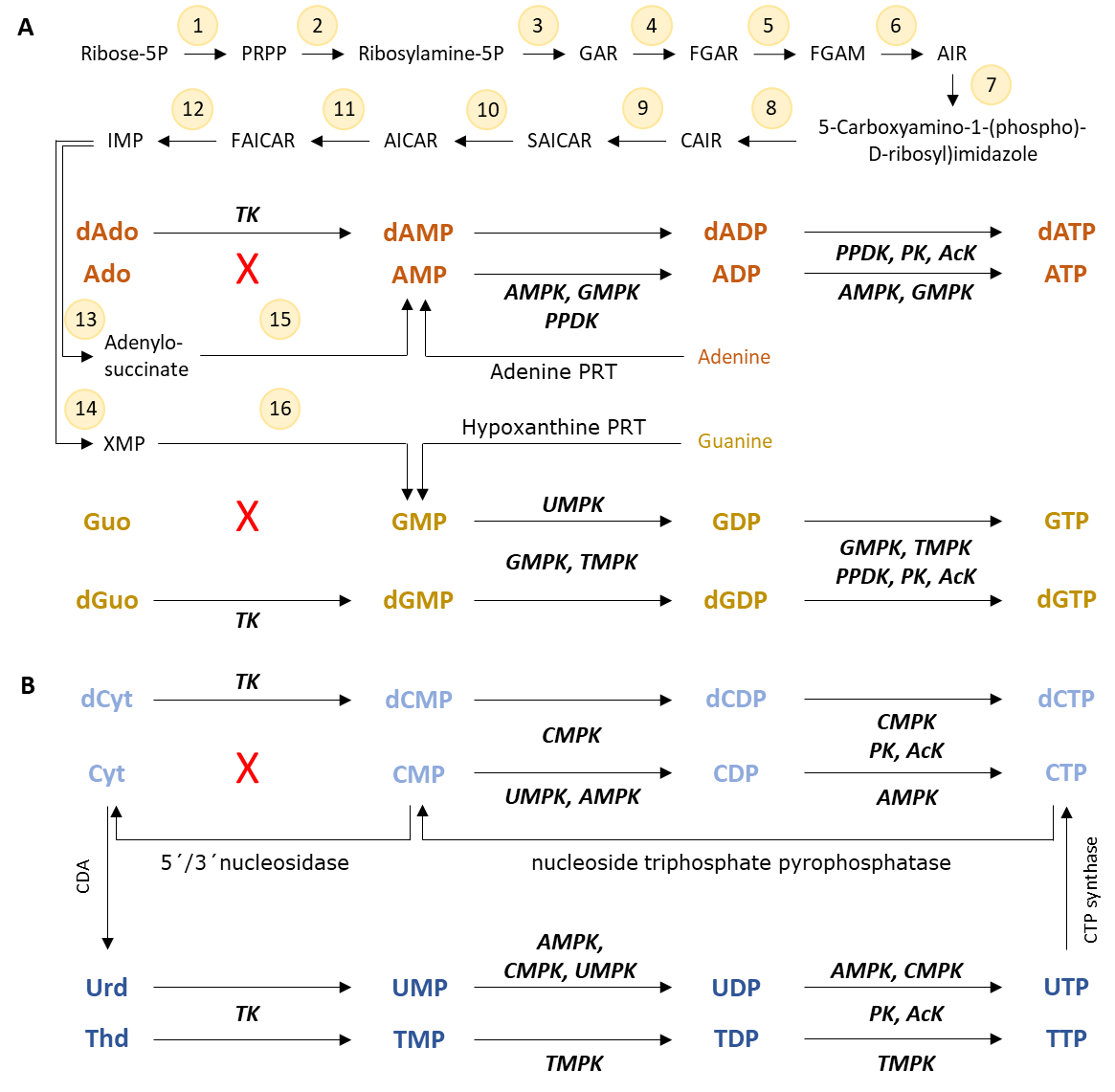


**Figure S1.** Synthesis of natural (deoxy)nucleotides in *T. maritima*. Purine (A) and pyrimidine (B) metabolism are shown. 1: ribose-phosphate pyrophosphokinase, 2: amidophosphoribosyltransferase, 3: phosphoribosylglycinamide synthetase, 4: phosphoribosylglycinamide formyltransferase 1, 5- phosphoribosylformylglycinamidine synthase PurS, 6: phosphoribosylformylglycinamidine cyclo-ligase, 7: 5-(carboxyamino)imidazole ribonucleotide synthase, 8: 5-(carboxyamino)imidazole ribonucleotide mutase, 9: phosphoribosylaminoimidazole-succinocarboxamide synthase, 10: adenylosuccinate lyase, 11: phosphoribosylaminoimidazolecarboxamide formyltransferase, 12: IMP cyclohydrolase, 13: adenylosuccinate synthase, 14: IMP dehydrogenase, 15: adenylosuccinate lyase, 16: GMP synthase (glutamine-hydrolysing), CDA: Cytidine deaminase, PRT- phosphoribosyltransferase


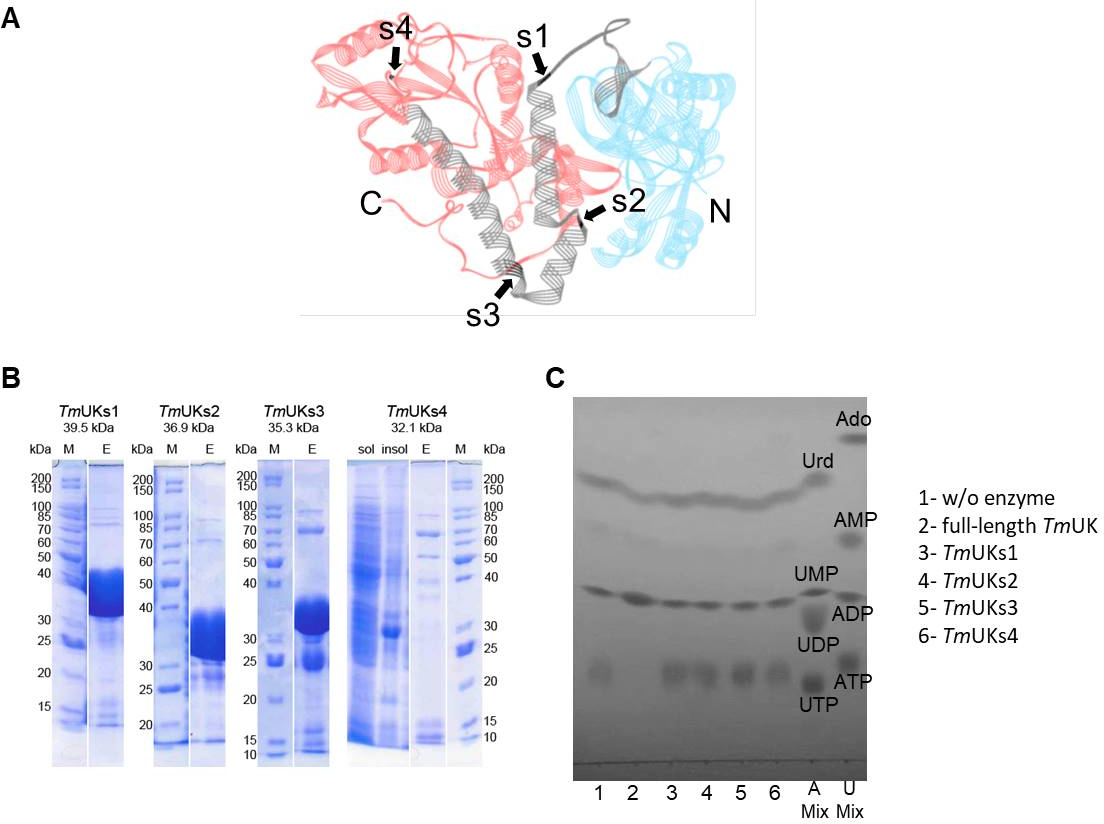


**Figure S2.** Construction and activity validation of truncated *Tm*UK variants.

A. Protein structure of *Tm*UK as predicted by Robetta’s RoseTTAFold algorithm. Light blue – predicted threonyl-tRNA synthetase domain, pink – predicted uridine kinase domain, grey – interdomain region. Arrows indicate starting amino acids of truncated *Tm*UK versions s1 (TmUKΔ1-226), s2 (TmUKΔ1-247), s3 (TmUKΔ1-261) and s4 (TmUKΔ1-290), respectively.

B. SDS-PAGE analysis of *Tm*UKs1-4 protein production. Proteins were purified from *E. coli* crude extracts via Ni-NTA chromatography. M – protein marker, E - elution fraction with highest concentration, sol – soluble fraction after protein extraction, insol – insoluble fraction after protein extraction. (C) Activity testing of *Tm*UKs1-4 towards uridine phosphorylation. Reaction mixtures contained 70 mM Tris-HCl (pH 7.6), 10 mM MgCl_2_, 100 mM KCl, 1 mM DTT, 1 mM uridine, 1.2 mM ATP and either no enzyme or 0.1 g L^-1^ of purified enzyme of full length *Tm*UK or the truncated version. Reactions were incubated at 55 °C for 19 h.


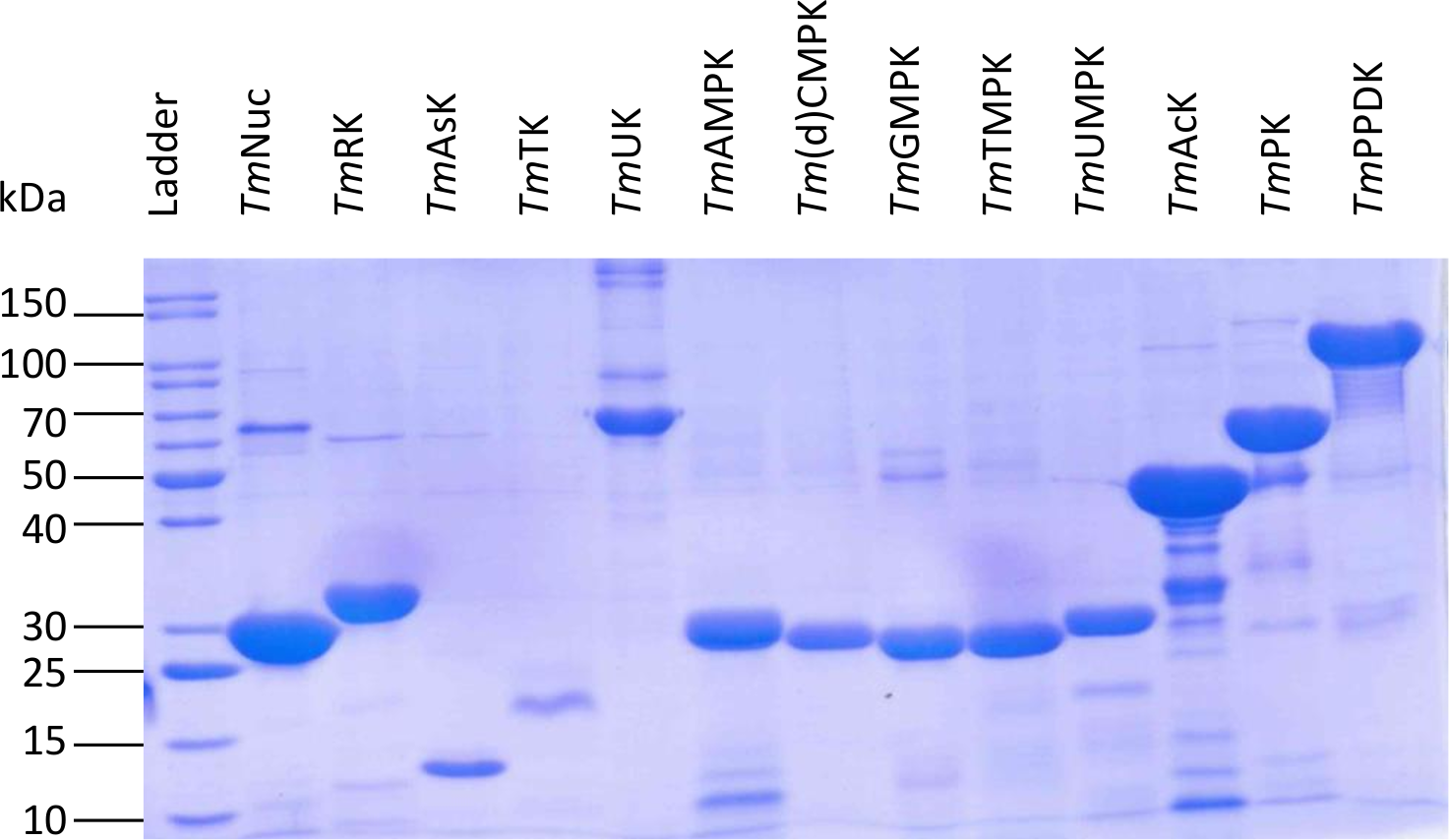


**Figure S3.** Purified enzymes of *T. maritima* putatively involved in the phosphorylation of nucleosides or nucleotides. Enzymes were expressed in EnPresso^®^ B medium, purified by affinity chromatography and dialyzed against a suitable buffer.


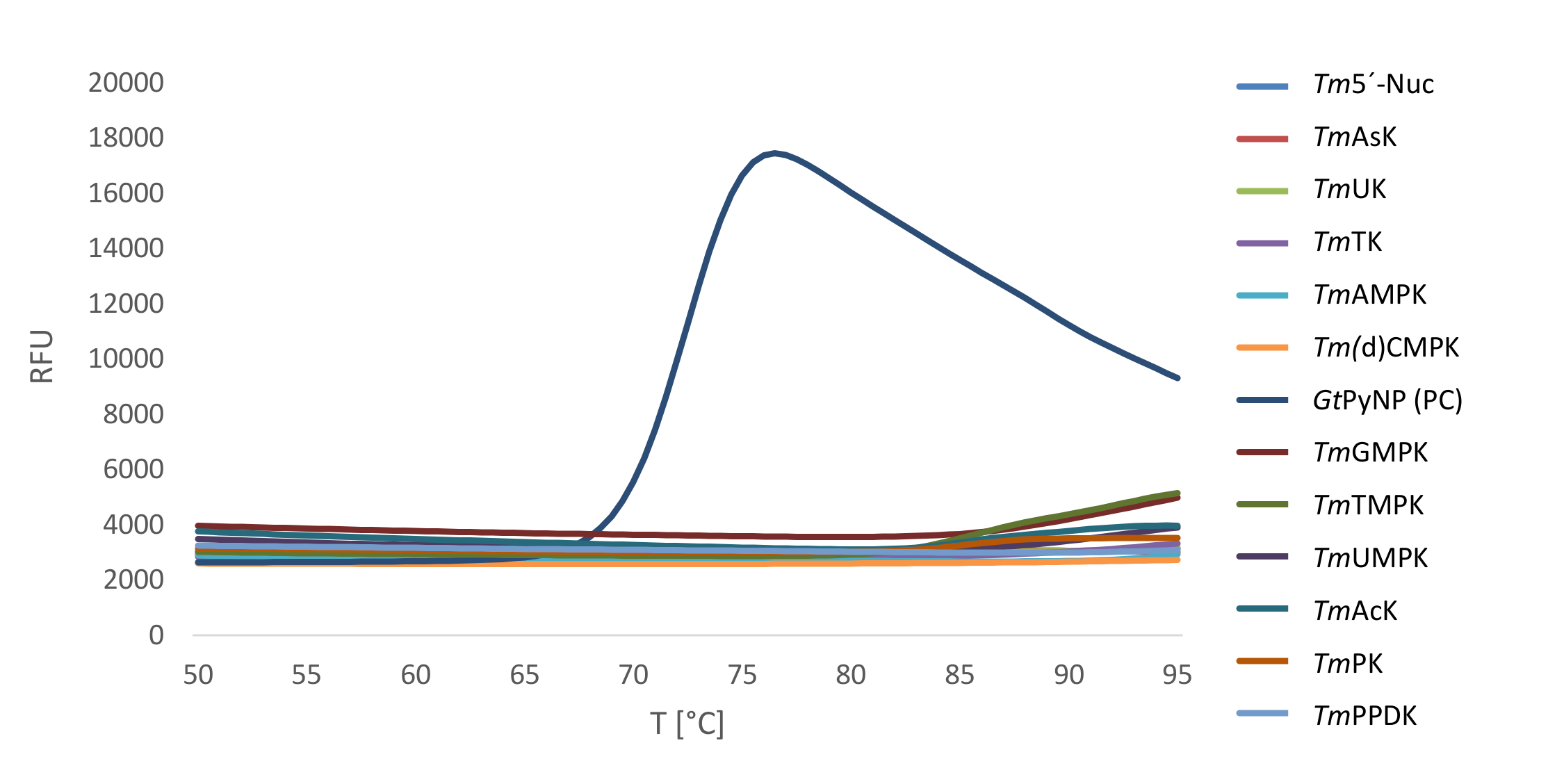


**Figure S4.** Melting point analysis for the 13 *T. maritima* enzymes putatively involved in the phosphorylation of nucleosides or nucleotides. Melting curve analysis was performed in Tris buffer (pH7) including 5 mM DTT. Pyrimidine nucleoside phosphorylase from *Geobacillus thermoglucosidasius* (*Gt*PyNP) was used as a positive control (PC), since a melting temperature of 77.5 °C was recently described for it [22].

**
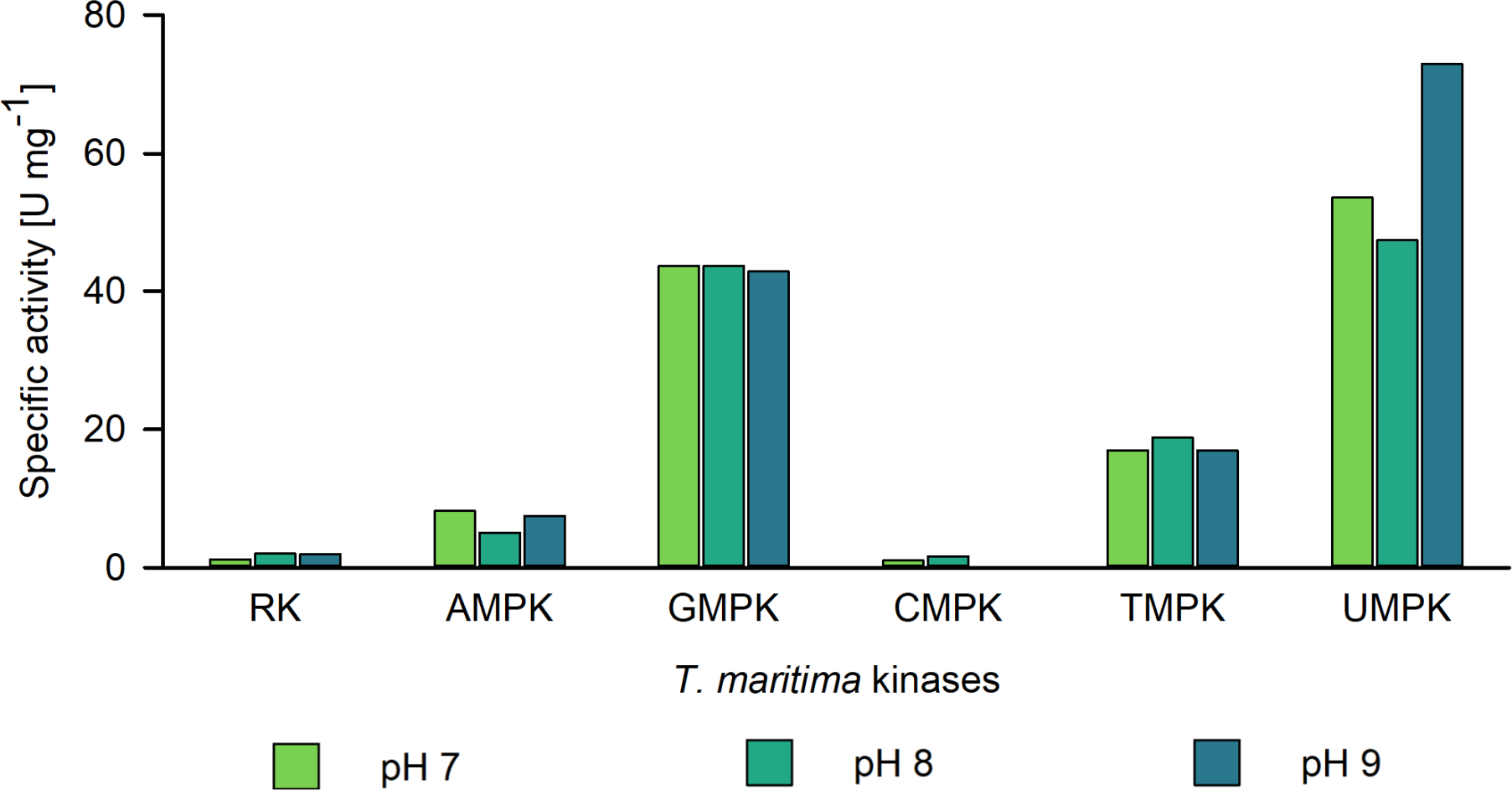
**

**Figure S5.** pH dependency of the RK and NMPKs of *T. maritima*. Specific activities were determined in 70 mM Tris buffer (pH 7, 8 or 9) including 2 mM MgCl_2_, 5 mM DTT, 50 mM KCl, 1 mM substrate (CMP: CMPK, dAMP: AMPK, GMP: GMPK, TMP: TMPK, UMP; UMPK, Urd: RK) and 1.2 mM ATP at 37 °C. Samples were stopped with cold deionized water and analyzed by the luminescent assay.


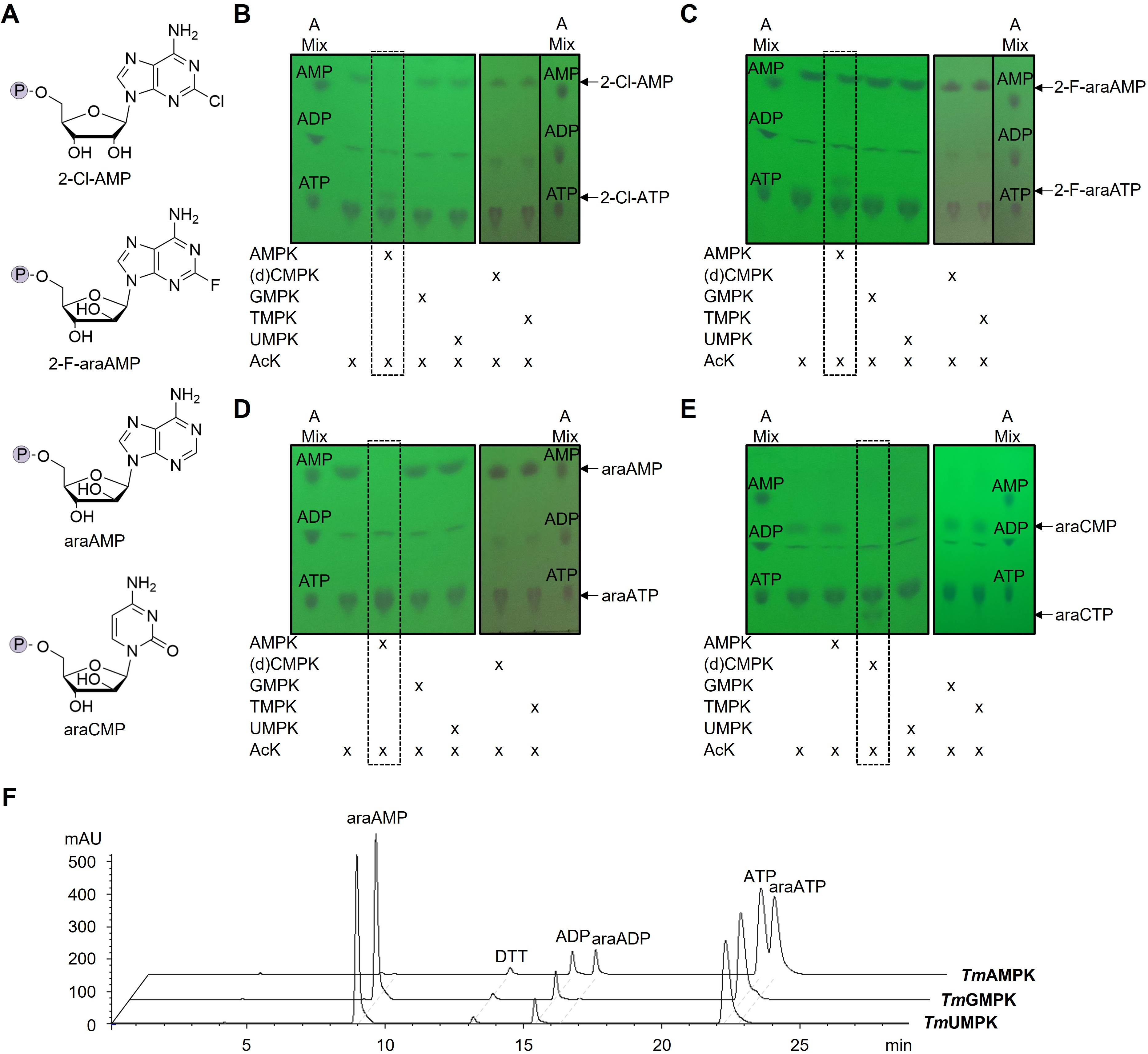


**Figure S6.** Enzymatic cascade reactions to produce sugar- or base modified 5´-NTPs starting from the NMP analog. The NMP analogs (A) 2-chloro-adenosine monophosphate (2-Cl-AMP, B), fludarabine phosphate (2-F-araAMP, C), vidarabine monophosphate (araAMP, D,E) and cytarabine monophosphate (araCMP) were tested as substrates with ATP as phosphate donor. An acetyl-phosphate ATP regeneration system using *Tm*AcK was included in the cascade reactions. Reactions were first analyzed by thin-layer chromatography (B-E). For araAMP as substrate reactions were also analyzed by HPLC as target compounds interfered with the phosphate donor and its degradation product (F). Reactions were performed with 1 mM NMP, 1.2 mM ATP, 5 mM AcetylP, 10 mM MgCl_2_, 5 mM DTT and 50 mM KCl in 70 mM Tris-HCl pH 7.6 for 19 h at 37 °C. As biocatalysts 0.04 mg mL^-1^ *Tm*NMPK and 0.02 mg mL^-1^ *Tm*AcK were applied. TLC plates were cut as indicated by the solid black line, and different plates were separated by a gap.

**
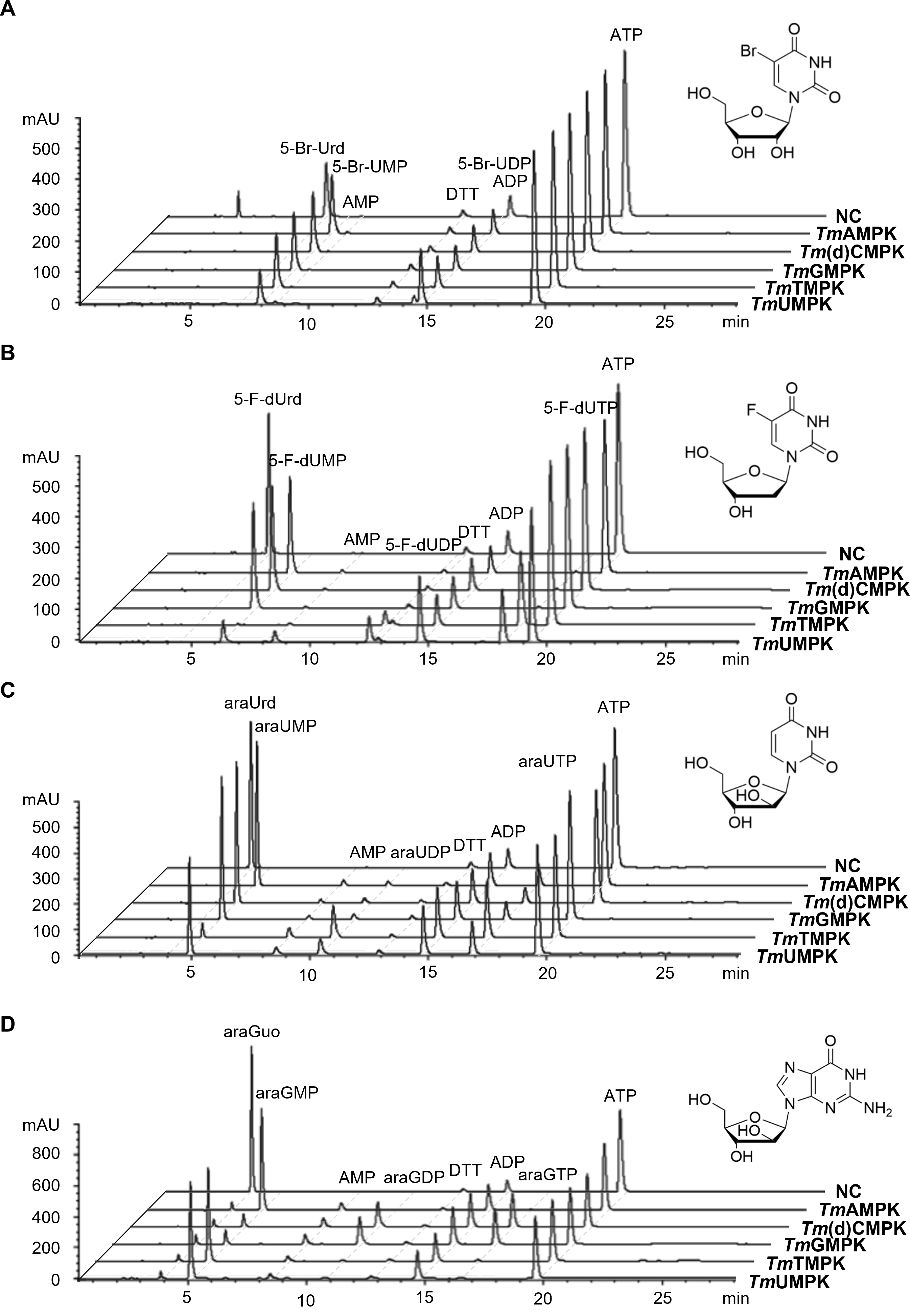
**

**Figure S7.** Enzymatic cascade reactions to produce sugar- or base modified 5´-NTPs starting from the nucleoside analog. 5-bromo-uridine (5-Br-Urd, A), 5-flouro-deoxyuridine (5-F-dU, B), arabinofuranosyluracil (araUrd, D) and arabinofuranosylguanine (araGuo, D) were applied as substrates and ATP as phosphate donor. An acetyl-phosphate ATP regeneration system using *Tm*AcK was included in the cascade reactions. Reactions were analyzed by HPLC. Reactions consisting of 1 mM nucleoside, 1.2 mM ATP, 5 mM AcetylP, 10 mM MgCl_2_, 5 mM DTT and 50 mM KCl in 70 mM Tris-HCl pH 7.6 were performed for 19 h at 37 °C. As biocatalysts 0.02 mg mL^-1^ *Hs*UCK2 (5-Br-Urd) or *Dm*dNK (rest), 0.04 mg mL^-1^ NMPK and 0.02 mg mL^-1^ *Tm*AcK were applied.

**References**

1. Hansen, T.; Schönheit, P. Sequence, Expression, and Characterization of the First Archaeal ATP-Dependent 6-Phosphofructokinase, a Non-Allosteric Enzyme Related to the Phosphofructokinase-B Sugar Kinase Family, from the Hyperthermophilic Crenarchaeote *Aeropyrum Pernix*. *Arch. Microbiol.* **2001**, *177*, 62–69, doi:10.1007/s00203-001-0359-1.

2. Hansen, T.; Arnfors, L.; Ladenstein, R.; Schönheit, P. The Phosphofructokinase-B (MJ0406) from *Methanocaldococcus Jannaschii* Represents a Nucleoside Kinase with a Broad Substrate Specificity. *Extremophiles* **2006**, *11*, 105, doi:10.1007/s00792-006-0018-1.

3. Aziz, I.; Bibi, T.; Rashid, N.; Aono, R.; Atomi, H.; Akhtar, M. A Phosphofructokinase Homolog from *Pyrobaculum Calidifontis* Displays Kinase Activity towards Pyrimidine Nucleosides and Ribose 1-Phosphate. *J. Bacteriol.* **2018**, *200*, doi:10.1128/JB.00284-18.

4. Elkin, S.R.; Kumar, A.; Price, C.W.; Columbus, L. A Broad Specificity Nucleoside Kinase from *Thermoplasma Acidophilum*. *Proteins Struct. Funct. Bioinforma.* **2013**, *81*, 568–582, doi:10.1002/prot.24212.

5. Li, Z.; Ning, X.; Zhao, Y.; Zhang, X.; Xiao, C.; Li, Z. Efficient One-Pot Synthesis of Cytidine 5’-Monophosphate Using an Extremophilic Enzyme Cascade System. *J. Agric. Food Chem.* **2020**, *68*, 9188–9194, doi:10.1021/acs.jafc.0c04055.

6. Blöndal, T.; Thorbjarnardóttir, S.H.; Kieleczawa, J.; Einarsson, J.M.; Hjörleifsdóttir, S.; Kristjánsson, J.K.; Eggertsson, G. Cloning, Sequence Analysis and Overexpression of a *Rhodothermus Marinus* Gene Encoding a Thermostable Thymidine Kinase. *FEMS Microbiol. Lett.* **1999**, *179*, 311–316, doi:10.1111/j.1574-6968.1999.tb08743.x.

7. Lutz, S.; Lichter, J.; Liu, L. Exploiting Temperature-Dependent Substrate Promiscuity for Nucleoside Analogue Activation by Thymidine Kinase from *Thermotoga Maritima*. *J. Am. Chem. Soc.* **2007**, *129*, 8714–8715, doi:10.1021/ja0734391.

8. Tomoike, F.; Nakagawa, N.; Kuramitsu, S.; Masui, R. A Single Amino Acid Limits the Substrate Specificity of *Thermus Thermophilus* Uridine-Cytidine Kinase to Cytidine. *Biochemistry* **2011**, *50*, 4597–4607, doi:10.1021/bi102054n.

9. Tomoike, F.; Nakagawa, N.; Kuramitsu, S.; Masui, R. Structural and Biochemical Studies on the Reaction Mechanism of Uridine-Cytidine Kinase. *Protein J.* **2015**, *34*, 411–420, doi:10.1007/s10930-015-9636-8.

10. Haney, P.J.; Stees, M.; Konisky, J. Analysis of Thermal Stabilizing Interactions in Mesophilic and Thermophilic Adenylate Kinases from the Genus *Methanococcus*. *J. Biol. Chem.* **1999**, *274*, 28453–28458, doi:10.1074/jbc.274.40.28453.

11. Konisky, J.; Michels, P.C.; Clark, D.S. Pressure Stabilization Is Not a General Property of Thermophilic Enzymes: The Adenylate Kinases of *Methanococcus Voltae*, *Methanococcus Maripaludis*, *Methanococcus Thermolithotrophicus*, and *Methanococcus Jannaschii*. *Appl. Environ. Microbiol.* **1995**, *61*, 2762–2764, doi:10.1128/aem.61.7.2762-2764.1995.

12. Rusnak, P.; Haney, P.; Konisky, J. The Adenylate Kinases from a Mesophilic and Three Thermophilic Methanogenic Members of the Archaea. *J. Bacteriol.* **1995**, *177*, 2977–2981, doi:10.1128/jb.177.11.2977-2981.1995.

13. Vieille, C.; Krishnamurthy, H.; Hyun, H.-H.; Savchenko, A.; Yan, H.; Zeikus, J.G. *Thermotoga Neapolitana* Adenylate Kinase Is Highly Active at 30 Degrees C. *Biochem. J.* **2003**, *372*, 577–585, doi:10.1042/BJ20021377.

14. Mega, R.; Nakagawa, N.; Kuramitsu, S.; Masui, R. The Crystal Structures of *Thermus Thermophilus* CMP Kinase Complexed with a Phosphoryl Group Acceptor and Donor. *PLoS One* **2020**, *15*, doi:10.1371/journal.pone.0233689.

15. Biswas, A.; Shukla, A.; Chaudhary, S.K.; Santhosh, R.; Jeyakanthan, J.; Sekar, K. Structural Studies of a Hyperthermophilic Thymidylate Kinase Enzyme Reveal Conformational Substates along the Reaction Coordinate. *FEBS J.* **2017**, *284*, 2527–2544, doi:10.1111/febs.14140.

16. Chaudhary, S.K.; Jeyakanthan, J.; Sekar, K. Cloning, Expression, Purification, Crystallization and Preliminary X-Ray Crystallographic Study of Thymidylate Kinase (TTHA1607) from *Thermus Thermophilus* HB8. *Acta Crystallogr. Sect. F* **2013**, *69*, 118–121, doi:10.1107/S1744309112050208.

17. Biswas, A.; Shukla, A.; Vijayan, R.S.K.; Jeyakanthan, J.; Sekar, K. Crystal Structures of an Archaeal Thymidylate Kinase from *Sulfolobus Tokodaii* Provide Insights into the Role of a Conserved Active Site Arginine Residue. *J. Struct. Biol.* **2017**, *197*, 236–249, doi:10.1016/j.jsb.2016.12.001.

18. Marco-Marín, C.; Escamilla-Honrubia, J.M.; Rubio, V. First-Time Crystallization and Preliminary X-Ray Crystallographic Analysis of a Bacterial-Archaeal Type UMP Kinase, a Key Enzyme in Microbial Pyrimidine Biosynthesis. *Biochim. Biophys. Acta - Proteins Proteomics* **2005**, *1747*, 271–275, doi:10.1016/j.bbapap.2004.11.010.

19. Jensen, K.S.; Johansson, E.; Jensen, K.F. Structural and Enzymatic Investigation of the *Sulfolobus Solfataricus* Uridylate Kinase Shows Competitive UTP Inhibition and the Lack of GTP Stimulation. *Biochemistry* **2007**, *46*, 2745–2757, doi:10.1021/bi0618159.

20. Takeishi, S.; Nakagawa, N.; Maoka, N.; Kihara, M.; Moriguchi, M.; Masui, R.; Kuramitsu, S. Crystallization and Preliminary X-Ray Diffraction Studies of Nucleoside Diphosphate Kinase from *Thermus Thermophilus* HB8. *Acta Crystallogr. D. Biol. Crystallogr.* **2003**, *59*, 1843–1845, doi:10.1107/s0907444903017712.

21. Pédelacq, J.-D.; Piltch, E.; Liong, E.C.; Berendzen, J.; Kim, C.-Y.; Rho, B.-S.; Park, M.S.; Terwilliger, T.C.; Waldo, G.S. Engineering Soluble Proteins for Structural Genomics. *Nat. Biotechnol.* **2002**, *20*, 927–932, doi:10.1038/nbt732.

22. Kaspar, F.; Wolff, D.S.; Neubauer, P.; Kurreck, A.; Arcus, V.L. PH-Independent Heat Capacity Changes during Phosphorolysis Catalyzed by the Pyrimidine Nucleoside Phosphorylase from *Geobacillus Thermoglucosidasius*. *Biochemistry* **2021**, *60*, 1573–1577, doi:10.1021/acs.biochem.1c00156.
